# Utility of genetic testing in suspected familial cranial diabetes insipidus

**DOI:** 10.1530/EDM-13-0068

**Published:** 2013-10-21

**Authors:** Ramesh Srinivasan, Stephen Ball, Martin Ward-Platt, David Bourn, Ciaron McAnulty, Tim Cheetham

**Affiliations:** 1 Department of Paediatric EndocrinologyRoyal Victoria Infirmary, Newcastle-upon-Tyne Hospitals NHS TrustNewcastle-upon-Tyne, NE1 4LPUK; 2 Department of EndocrinologyRoyal Victoria InfirmaryNewcastle-upon-Tyne, NE1 4LPUK; 3 The Medical School, Newcastle UniversityNewcastle, NE24HHUK; 4 Ward 35 Royal Victoria InfirmaryNewcastle-upon-Tyne NE1 4LPUK; 5 The Institute of Genetic Medicine, Newcastle University International Centre for Life Central ParkwayNewcastle-upon-Tyne, NE1 3BZUK

## Abstract

**Aim:**

Differentiating familial cranial diabetes insipidus (CDI) from primary polydipsia can be difficult. We report the diagnostic utility of genetic testing as a means of confirming or excluding this diagnosis.

**Patient and methods:**

The index case presented at 3 months with polydipsia. He was diagnosed with familial CDI based on a positive family history combined with what was considered to be suspicious symptomatology and biochemistry. He was treated with desmopressin (DDAVP) but re-presented at 5 months of age with hyponatraemia and the DDAVP was stopped. Gene sequencing of the vasopressin gene in father and his offspring was undertaken to establish the underlying molecular defect.

**Results:**

Both father and daughter were found to have the pathogenic mutation c.242T>C (p.Leu81Pro) in exon 2 of the *AVP* gene consistent with a diagnosis of familial diabetes insipidus. The index case did not have the pathogenic mutation and the family could be reassured that he would not require intervention with DDAVP.

**Conclusions:**

Gene sequencing of *AVP* gene can have a valuable role in predicting whether or not a child is at risk of developing CDI in future. This can help to prevent family uncertainty and unnecessary treatment with its associated risks.

**Learning points:**

- Differentiating patients with familial cranial diabetes insipidus from those with primary polydipsia is not always straightforward.
- Molecular genetic analysis of the vasopressin gene is a valuable way of confirming or refuting a diagnosis of familial CDI in difficult cases and is a valuable way of identifying individuals who will develop CDI in later childhood. This information can be of great value to families.

## Background

Familial cranial diabetes insipidus (FCDI) is a rare inherited disorder that may have an autosomal dominant inheritance pattern. Polyuria and polydipsia typically develop in childhood due to a progressive decline in AVP secretion and selective degeneration of AVP-producing neurons (1). Differentiating evolving cranial diabetes from the more common cause of excessive fluid intake, primary polydipsia, is not always straightforward. In our experience, this can be particularly difficult in the early phase of cranial diabetes insipidus (CDI) when AVP secretion is declining. Here, we highlight the diagnostic utility of *AVP* gene sequencing in the context of a child with suspected autosomal dominant cranial diabetes insipidus who became hyponatraemic while on desmopressin (DDAVP) medication.

## Case presentation

The index case was thought to be drinking excessively by his parents at 3 months of age. Father had been diagnosed with CDI in adolescence and was on regular DDAVP therapy under the care of the adult endocrine team. The child's older female half-sibling had been diagnosed with CDI at 8 years of age and was stable on DDAVP therapy.

The index case was assessed in clinic and the combination of a positive family history, suspected polyuria despite loose stools and poor feeding combined with a urine osmolality of 53 mOsm/kg at the time of a serum sodium of 140 mmol/l and serum osmolality of 284 mOsm/kg was thought to be indicative of the child-developing CDI like his father. He was therefore commenced on nasal DDAVP in a dose of 5 μg b.d. The DDAVP dose was subsequently increased up to 15 μg b.d. because of ongoing concerns about an excessive thirst and urine output.

The infant was admitted to hospital at 5 months of age with a history of poor feeding, diarrhoea and vomiting. Investigations at the time of his admission revealed a sodium of 120 mmol/l in the presence of a low serum creatinine (Table 1). The admitting team initially wondered about the possibility of inter-current infection but the diagnosis was revised to include one of water intoxication and the DDAVP was stopped. In light of the fact that the biochemistry at the time of DDAVP initiation had not been diagnostic of CDI, it was felt appropriate to conduct further investigations including a water deprivation test.

**Table 1:** Resolution of hyponatraemia following cessation of DDAVP

**Day and time**	**Serum sodium** (mmol/l)	**Serum osmolality** (mOsm/kg)	**Serum creatinine** (mmol/l)	**Urine osmolality** (mOsm/kg)	**Urine sodium** (mmol/l)
Day 1 22.00	120		<20	62	<10
Day 2 06.00	125	257	<20	63	<10
Day 2 14.00	127		17	72	
Day 3 08.00	127		25		
Day 5 11.00	136	283	<20	563	
Day 5 13.00	136	282	22		

Forty-eight hours after admission, the infant's sodium level had risen to 127 mmol/l (Table 1). Five days after admission, he underwent an eight-and-a-half-hour fast, at the end of which his sodium level was 136 mmol/l and his urine osmolality level was 563 mOsm/l. He had not received any DDAVP for more than 4 days. The patient was discharged with advice regarding offering water intermittently in addition to milk and solid feeds.

## Investigation

### Genetic analysis

In discussion with the family, it was decided to investigate the father with a view to establishing the underlying molecular defect in the vasopressin gene. We agreed that it would then be appropriate to establish whether his offspring were affected by the same molecular defect.

PCR of the three *AVP* coding exons and flanking intronic sequences was performed separately using the primers mentioned in Table 2. Primers were tagged with universal primers UniSeq. The reaction contained ∼150 ng genomic DNA, 1U Immolase Taq (Bioline Reagents Ltd, London, UK), 20 pmol of each primer, 200 μM dNTPs, 1.5 mM MgCl_2_, 2× ImmoBuffer (Bioline) and 15% DMSO in a total volume of 20 μl.

**Table 2:** Primers used in vasopressin gene analysis

**Exon**	**Forward sequence**	**Reverse sequence**
1	GAACACCTGCGGACATAAATAG	CTAAAGGCTACCACCACCCATG
2	AGCCCTGGACCCCAGCATC	CAGCCCCCACCCCGCCGCA
3	GTTTGCTGCAACGACGGTGC	GAGGCCGTGCATTGGCGGAG

The PCR conditions consisted of an initial denaturing step at 95 °C for 5 min followed by 32 cycles of denaturation (1 min at 95 °C), annealing (1 min at 60 °C for exon 1, 1 min at 65 °C for exons 2 and 3) and extension (1 min at 72 °C) with a final extension step at 72 °C for 10 min.

### DNA sequencing

Amplified targets were purified using AmPure (Agencourt, Beverly, MA, USA) and directly sequenced in the forward and reverse directions using ABI Big Dye Terminator v3.1 cycle sequencing kit (Applied Biosystems). Sequencing products were purified using CleanSDefault (Agencourt) before being read using an ABI 3130XL DNA analyser (Applied Biosystems). Sequence traces were analysed using Mutation Surveyor v3.97 (Soft Genetics, State College, PA, USA).

## Treatment

The result of genetic testing was available within 4 weeks. Both father and daughter were found to have the pathogenic mutation c.242T>C (p.Leu81Pro) in exon 2 of the *AVP* gene consistent with a diagnosis of familial diabetes insipidus. This change had been reported previously in another kindred (2).

Genetic testing of the index case showed that he did not have the pathogenic mutation and hence was not at significant risk of developing CDI.

## Outcome and follow-up

The child was discharged without further follow-up.

## Discussion

This case highlights the valuable role of genetic testing in the assessment and management of children with suspected CDI. A child with a family history of autosomal dominant CDI and who is thought to be polyuric with a low urine osmolality at the time of illness will have a relatively high likelihood of being similarly affected. Parental concerns about evolving CDI may also have some bearing on the assessment and management of a clinical scenario like this.

However, it is important to highlight the fact that the child's biochemistry was not diagnostic of CDI and the development of hyponatraemia reflected water intoxication following DDAVP medication. This case therefore highlights the importance of using robust clinical, biochemical and potentially molecular genetic criteria before commencing DDAVP therapy.

Familial CDI is rare, accounting for about 5% of all cases. The clinical expression of familial autosomal dominant disorder does not usually occur in infancy but instead at around 5–10 years (3).

Assessment of the child with suspected DI may involve water deprivation testing, although this test is unpopular with children, parents and health professionals and does not necessarily provide a definitive diagnosis.

Genetic analysis of patients where there is a relevant family history can therefore be undertaken in early life following discussion with the family (4). This can be used to identify children who are likely to develop CDI and will help parents and health professionals to monitor (or not monitor) potential symptoms and signs as the child matures (5) (6). In a cost-conscious health service, it should also be stressed that the cost of genetic analysis (100 pounds for the index case and 70 pounds for each additional family member) is much cheaper than the cost of a water deprivation test (300–400 pounds).

In conclusion, we have shown that genetic analysis of the *AVP* gene can have a valuable role in identifying whether or not a child with a family history of CDI will develop or is at risk of developing CDI in future.

## Patient's perspective

Due to the misdiagnosis, I nearly lost my son. Obviously, if a genetic test had been carried out prior to this, this would not have happened and I would not have been in this situation. Luckily, he is now well with no problems.

## Patient consent

Written informed consent has been obtained from the patient's parents.

## Author contribution statement

R Srinivasan contributed to literature search and awriting; D Bourn and C McAnulty contributed to literature search,writing and genetic testing; S Ball and M Ward-Platt contributed to writing; and T Cheetham contributed to the literature search, writing, and to the supervision and patient management.
